# Optimization of Ultrasound-Assisted Extraction of Antioxidant Polyphenols from the Seed Coats of Red Sword Bean (*Canavalia gladiate* (Jacq.) DC.)

**DOI:** 10.3390/antiox8070200

**Published:** 2019-06-28

**Authors:** Yue Zhou, Xiao-Yu Xu, Ren-You Gan, Jie Zheng, Ya Li, Jiao-Jiao Zhang, Dong-Ping Xu, Hua-Bin Li

**Affiliations:** 1 Guangdong Provincial Key Laboratory of Food, Nutrition and Health, Department of Nutrition, School of Public Health, Sun Yat-Sen University, Guangzhou 510080, China; 2 Department of Food Science & Technology, School of Agriculture and Biology, Shanghai Jiao Tong University, Shanghai 200240, China

**Keywords:** *Canavalia gladiata*, bean coat, food waste, ultrasound-assisted extraction, antioxidant, polyphenols

## Abstract

The seed coat of red sword bean (*Canavalia gladiata* (Jacq.) DC.) is rich in antioxidant polyphenols. It is often discarded as a byproduct with the consumption of red sword bean, since it is very thick and not consumed by people. The aim of this study was to develop an ultrasound-assisted extraction method to extract natural antioxidants from the seed coats. The extraction process was optimized by using response surface methodology. After the single-factor experiments, three key factors, including ethanol concentration, liquid/solid ratio, and extraction time, were selected and their interactions were studied using a central composite design. The optimal extraction condition was 60.2% hydroethanol, a liquid/solid ratio of 29.3 mL/g, an extraction time of 18.4 min, an extraction temperature of 50 °C, and ultrasound power of 400 W. Under the optimal conditions, antioxidant activity of the extract was 755.98 ± 10.23 μmol Trolox/g dry weight (DW), much higher than that from maceration (558.77 ± 14.42 μmol Trolox/g DW) or Soxhlet extraction (479.81 ± 12.75 μmol Trolox/g DW). In addition, the main antioxidant compounds in the extract were identified and quantified by high-performance liquid chromatography–diode array detection–tandem mass spectrometry (HPLC–DAD–MS/MS). The concentrations of digalloyl hexoside, methyl gallate, gallic acid, trigalloyl hexoside, and digallic acid were 15.30 ± 0.98, 8.85 ± 0.51, 8.76 ± 0.36, 4.27 ± 0.21, and 2.89 ± 0.13 mg/g DW. This study provides an efficient and green extraction method for the extraction of natural antioxidants from the bean coat of red sword bean. The extract of antioxidants might be added into functional foods or nutraceuticals with potential beneficial functions.

## 1. Introduction

Oxidative stress deregulates various cellular functions and can lead to a number of pathological changes, such as aging, cancer, neurodegenerative diseases, and cardiovascular diseases [1]. Antioxidants can combat oxidative stress, and protect against these diseases [2]. Therefore, antioxidants, especially natural ones, draw great research interest in the food, pharmaceutical, and cosmetic industries.

Recent studies suggested that legumes are abundant in natural antioxidants, and the 1,1-diphenyl-2-picrylhydrazyl (DPPH) radical-scavenging activity of sword bean was higher than that of α-tocopherol [3,4]. The nutritious seeds of red sword bean (RSB, *Canavalia gladiata* (Jacq.) DC.) can be used to make soups and stews, and they are also used in traditional medicine [5]. Recently, our study found that RSB had the highest antioxidant property among 42 edible beans, and such activity was mainly attributed to its seed coat, which is often discarded as a byproduct when consuming the bean [6,7,8]. In addition, RSB has promising pharmaceutical potential, due to its anti-angiogenic, hepatoprotective, and anti-inflammatory activities [9,10,11]. The seed coat of RSB can be a good source of natural antioxidants, which might be used to prevent and treat several diseases induced by oxidative stress [8]. Therefore, it is necessary to find an efficient extraction method to recover natural antioxidants from the seed coat of RSB to make the best use of this byproduct.

In past decades, the shortcomings of conventional extraction methods, such as the use of toxic organic solvents, a long extraction time, and low efficiency, led to increasing demand for efficient, sustainable, and green extraction techniques. To meet this need, several new extraction methods were established, such as ultrasound-assisted extraction (UAE), supercritical fluid extraction, and microwave-assisted extraction [12,13,14,15]. Among those extraction methods, UAE has several prominent advantages. The whole extraction process can be completed within minutes, with high reproducibility and reduced use of toxic organic solvents, as well as energy. Also, UAE is easy to manipulate without complex and expensive equipment [16]. In the extraction of plant products, the application of ultrasound causes the propagation of high-frequency ultrasonic waves that result in cavitation, which can break the plant tissues and enhance the penetration of solvents and mass transfer [17,18]. Several studies reported that UAE can be a powerful and efficient method for the extraction of natural antioxidants from plant materials [19]. To our knowledge, there is no study regarding the optimal extraction of antioxidants from the seed coats of RSB using UAE. Therefore, this study aimed to develop a UAE method to recover antioxidants from the seed coat byproduct of RSB.

There is no standard protocol for UAE, and several parameters, such as the solvent concentration, liquid/solid ratio (LS ratio), extraction time, ultrasound power, and extraction temperature, may significantly affect the extraction efficiency. Therefore, the determination and the optimization of key parameters are important for maximizing the yield of antioxidants. Response surface methodology (RSM) is commonly used to optimize the complex extraction conditions, and it can simultaneously evaluate the effects of different parameters, as well as their interactions [20,21]. In this study, the effects of several parameters on the extraction efficiency were studied, and the parameters were optimized, including solvent concentration, LS ratio, extraction time, ultrasound power, and extraction temperature. Furthermore, the main antioxidant compounds in the extract were identified and quantified by high-performance liquid chromatography–diode array detection–tandem mass spectrometry (HPLC–DAD–MS/MS). In addition, the extraction efficiency of UAE was compared with two traditional extracting methods, namely maceration and Soxhlet extraction. 

## 2. Materials and Methods 

### 2.1. Chemicals and Reagents

Gallic acid, 2,2′-azinobis(3-ethyl-benzothiazoline-6-sulfonic acid) diammonium salt (ABTS), and 6-hydroxy-2,5,7,8-tetramethylchromane-2-carboxylic acid (Trolox) were obtained from Sigma-Aldrich (St. Louis, MO, USA). Methyl gallate was purchased from Yuanye Bio-tech Co., Ltd. (Shanghai, China). Potassium persulfate, ethanol, and methanol were provided by Kelong Chemical Factory (Chengdu, China). All chemicals were of analytical grade. Deionized water was employed in all experiments.

### 2.2. Sample Preparation

RSB was bought at the local market in Guangzhou, China, and identified by Dr. Sha Li from the School of Chinese Medicine, University of Hong Kong. The voucher specimen was preserved in the School of Public Health, Sun Yat-Sen University (No. RSB-20170108-01). The seed coats of RSB were manually removed, dried in the oven at 40 °C for 24 h, and ground into fine powder, before being kept at 4 °C until used.

### 2.3. Experimental Design

Single-factor experimental optimization was conducted to investigate effects of different extraction parameters on the extraction efficiency, including the concentration of ethanol (20, 30, 40, 50, 60, 70, and 80%), LS ratio (10, 15, 20, 25, 30, 35, and 40 mL/g), extraction time (0, 5, 15, 20, 25, and 30 min), extraction temperature (30, 40, 50, 60, 70, and 80 °C), and ultrasound power (200, 300, 400, 500, 600, 700, and 800 W). According to the results of single-factor optimization, three key variables were selected for RSM optimization. A five-level three-factor central composite design was adopted to investigate the interactions of these variables on the extraction efficiency. Each parameter was coded at five levels (−1.68, −1, 0, 1, and 1.68). The design consisted of 20 experimental runs. The data were fitted into a second-order polynomial model as follows:

Y = β_0_ + ∑β_i_X_i_ + ∑β_ii_ X_i_^2^ + ∑β_ij_ X_i_ X_j_, (1)

where Y represents the response, X_i_ and X_j_ stand for different independent variables, and β_0_, β_i_, β_ii_, and β_ij_ are the intercept, linear, quadratic, and interaction regression coefficients, respectively.

### 2.4. Extraction Process

#### 2.4.1. UAE procedure

The powdered sample (0.500 g) was accurately weighed, mixed with respective solvents, and then extracted using an ultrasonic bath (Kj1012B; Kejin Ultrasonic Instrument Factory, Guangzhou, China). After the ultrasound treatment, the mixture was centrifuged (4200× *g*, 30 min); then, the supernatant was collected and kept at 4 °C for further experiments.

#### 2.4.2. Maceration

The powdered sample (0.500 g) was accurately weighed, mixed with the extraction solvent (60.2% hydroethanol, and LS ratio of 29.3 mL/g), and then extracted using a shaking water bath at 30 °C for 24 h. After that, the mixture was centrifuged (4200× *g*, 30 min), and the supernatant was collected for further experiments.

#### 2.4.3. Soxhlet Extraction

The Soxhlet extraction method was carried out according to the literature with a slight modification [22]. The powdered sample (0.500 g) was accurately weighed, wrapped in Whatman filter paper, and extracted in the Soxhlet extractor using 200 mL of 60.2% hydroethanol (*v*/*v*) as the extraction solution at 95 °C for 4 h. The mixture was then centrifuged (4200× *g*, 30 min), and the supernatant was collected for further experiments.

### 2.5. Determination of Antioxidant Capacity

Several methods were established to evaluate the antioxidant capacity of natural extracts in vitro. In general, the extraction procedure can be monitored using different evaluation methods of antioxidant capacity, because these methods are usually highly correlated with each other [3,23,24]. The Trolox equivalent antioxidant capacity (TEAC) assay is often chosen to evaluate the antioxidant capacity of natural extracts due to its simplicity, repeatability, and rapidity [25]. Therefore, the TEAC assay was conducted in this study to evaluate the antioxidant capacity as previously described [22]. ABTS stock solution was prepared by using a 1:1 ratio (*v*/*v*) of ABTS (7 mmol/L) and potassium persulfate (2.45 mmol/L), and was stored for 16 h in the dark before use. The ABTS working solution was prepared by dilution of the ABTS stock solution to an absorbance of about 0.700 ± 0.05 at 734 nm. For testing, the ABTS working solution (3.8 mL) was mixed with a properly diluted sample (100 μL) and the mixture was incubated at room temperature (25 °C) for 6 min in the dark. After that, the absorbance of the mixture was determined at 734 nm using a spectrophotometer (Model 722, Shanghai precision instrument Co., Ltd., Shanghai, China). Trolox was used as the reference standard, and the results were presented as µmol Trolox/g dry weight (DW) of sample powders.

### 2.6. Determination of Total Phenolic Contents

The determination of total phenolic contents was conducted using the Folin–Ciocalteu method as described in the literature [26]. Briefly, Folin–Ciocalteu reagent (0.2 mol/L, 2.5 mL) was mixed with the properly diluted sample (0.50 mL), and the mixture was incubated for 4 min at room temperature (25 °C), followed by adding the saturated sodium carbonate solution (2 mL); this mixture was incubated at room temperature (25 °C) for 2 h in the dark. The absorbance of the mixture was determined at 760 nm. Gallic acid was used as a reference standard, and the results were presented as mg gallic acid equivalent (mg GAE)/g DW of sample powders.

### 2.7. Determination of Total Flavonoid Contents

The determination of total flavonoid contents was conducted using the AlCl_3_-based colorimetric method as described in the literature [27]. Briefly, the properly diluted sample (0.5 mL) was added to 95% ethanol (*v*/*v*, 1.5 mL), a 10% AlCl_3_ solution (*w*/*v*, 0.1 mL), and deionized water (2.8 mL), and then the mixture was incubated at room temperature (25 °C) for 30 min. After that, the absorbance of the mixture was determined at 415 nm. Catechin was used as a reference standard, and the results were presented as mg catechin equivalent (mg CE)/g DW of sample powders.

### 2.8. Identification and Quantification of Main Antioxidant Polyphenols based on HPLC–DAD–MS/MS 

The main antioxidant polyphenols in RSB seed coats obtained under the optimal extraction conditions were identified and quantified by HPLC–DAD–MS/MS according to a previous study [8] with some modifications. In brief, sample separation was conducted with an Agilent 1290 Infinity HPLC system composed of an auto-sampler, a binary pump, a diode array detector, and an MS/MS component. A Symmetry Shield^TM^ RP18 column (150 × 2.1 mm, 3.5 μm) (Waters, USA) with the gradient elution, including solution A (0.1% formic acid–water solution) and solution B (0.1% formic acid–methanol solution), was used. The program was set as follows: 0 min, 5% B; 15 min, 20% B; 40 min, 35% B; 60 min, 50% B; 65 min, 55% B; 70–75 min, 95% B. Injection volume was 5 μL, flow rate was 0.2 mL/min, and the detection wavelength was 280 nm for detecting gallic acid and its derivatives. The main phenolic compounds were verified and quantified by comparing the retention time and peak area with the respective standard. Since only the standards of gallic acid and methyl gallate were available, the contents of other gallic acid derivatives were calculated based on gallic acid equivalent.

### 2.9. Statistical Analysis

All experiments were conducted in triplicate, and the results were presented as means ± standard deviation (SD). One-way ANOVA plus a post hoc Turkey test was performed to analyze the differences of data (*n* > 2). The RSM was analyzed with Design Expert (version 8.0.6, Stat-Ease, Minneapolis, MN, USA), and the statistical analysis was carried out with SPSS 19.0 (IBM SPSS Statistics, IBM Corp, Somers, NY, USA) or Excel 2016 (Microsoft, Redmond, WA, USA). Statistical significance was defined at *p* < 0.05.

## 3. Results and Discussion

### 3.1. Results from Single-Factor Experiments

During the process of UAE, many parameters could affect the extraction efficiency, such as the concentration of solvent, LS ratio, extraction time, ultrasound power, and extraction temperature. Therefore, the single-factor experiments were carried out to determine the parameters that should be considered for further optimization design.

#### 3.1.1. The Effect of Ethanol Concentration

**Figure 1:**
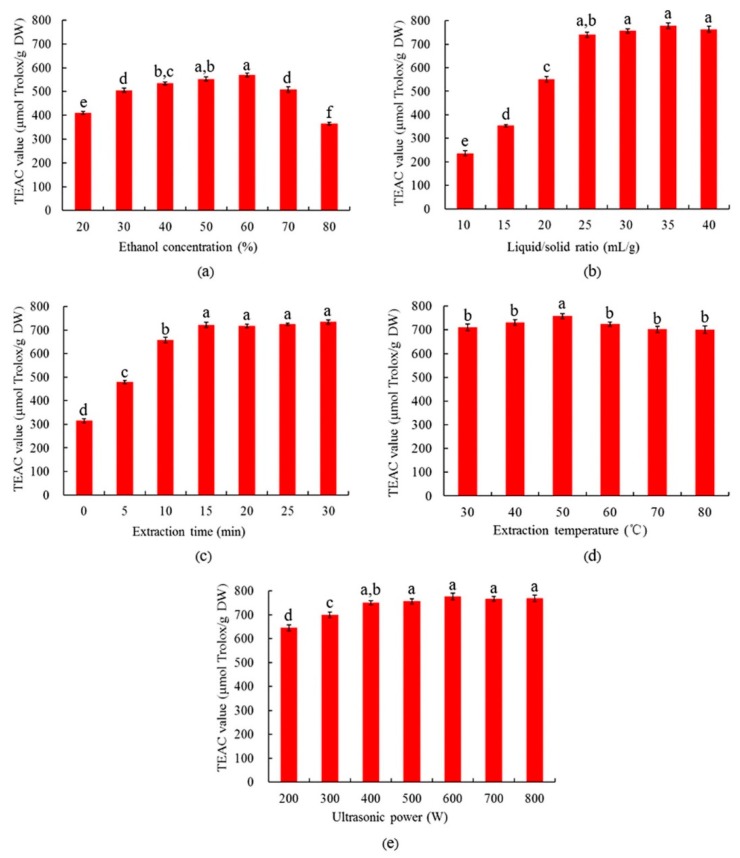
Effects of different extraction parameters, including (**a**) ethanol concentration, (**b**) liquid/solid (LS) ratio, (**c**) extraction time, (**d**) extraction temperature, and (**e**) ultrasound power, on Trolox equivalent antioxidant capacity (TEAC) values. Different lowercase letters indicate statistical significance at *p* < 0.05.

Ethanol–water mixtures are common solvents for extracting antioxidants from plant samples [28]. In addition, ethanol is safe and environmentally friendly. Therefore, an ethanol–water mixture was selected as the solvent in this study. The effect of different ethanol concentrations (20, 30, 40, 50, 60, 70, and 80%) on the extraction efficiency was evaluated. Meanwhile, other extraction conditions were controlled as follows: LS ratio at 1:20, extraction time at 20 min, extraction temperature at 30 °C, and ultrasound power at 400 W. The antioxidant activity of the extracts was measured by the TEAC assay. As shown by Figure 1a, an increase in TEAC value was observed when the concentration of ethanol increased from 20% to 60%, and the highest antioxidant activity was observed at 60% ethanol. However, a further increase in ethanol concentration decreased antioxidant activity. The change in the extraction efficiency could be attributed to the fact that different ethanol concentrations changed the polarity of solvent, and adding water to ethanol could increase the polarity of solvent [25]. According to the principle of “like dissolves like”, the solubility of target compounds varies with the polarity of the solvent [29]. Our results indicated that the antioxidant compounds in the extracts of RSB seed coats had the best solubility when ethanol concentration was 60%; therefore, a 60% ethanol solution was selected for subsequent studies. 

#### 3.1.2. The Effect of Liquid/Solid Ratio

The effect of LS ratio ranging from 10−40 mL/g on the extraction efficiency was determined, with other parameters remaining constant as follows: ethanol at 60%, temperature at 30 °C, extraction time at 20 min, and ultrasound power at 400 W. As shown in Figure 1b, when LS ratio increased from 10 to 25 mL/g, antioxidant activity rapidly increased. However, the antioxidant activity was not significantly changed at ratios higher than 25 mL/g. The reason might be that a high LS ratio could create a high concentration gradient between the solvent and the material, which promoted mass transfer until the latter reached its maximum [30]. However, an over-high LS ratio might also decrease the dispersion of ultrasound energy. Also, the use of a large amount of organic solvent would increase the extraction cost, as well as the amount of waste. Thus, an LS ratio of 25 mL/g was chosen as the optimal ratio.

#### 3.1.3. The Effect of Extraction Time 

The extraction time plays an important role in the extraction process. The extraction time ranging from 0 to 30 min was investigated in this study, with other parameters kept constant as follows: ethanol at 60%, LS ratio at 25 mL/g, extraction temperature at 30 °C, and ultrasound power at 400 W. As shown in Figure 1c, the extraction time had a markedly positive effect on the extraction of antioxidants during the first 15 min. The ultrasound treatment of 15 min was adequate for extracting most antioxidants from the RSB seed coats. Additionally, it was reported that a long ultrasonic treatment could induce the degradation of antioxidants, leading to a decreased TEAC value [31,32]. Therefore, 15 min of ultrasound time was chosen for subsequent studies.

#### 3.1.4. The Effect of Extraction Temperature

Temperatures ranging from 30 to 80 °C were selected for studying the effect of different temperature on the extraction efficiency. Meanwhile, other parameters were kept constant as follows: ethanol concentration at 60%, LS ratio at 25 mL/g, extraction time at 15 min, and ultrasound power at 400 W. As shown in Figure 1d, the temperature had little effect on the extraction efficiency. The antioxidant activity slightly increased from 30 °C to 50 °C, and then slightly decreased. The results indicated that the major proportion of antioxidants could be extracted at a relatively low temperature, and a high temperature might induce the degradation of certain antioxidants, which was in agreement with previous studies [33]. As a result, 50 °C was chosen for subsequent studies. 

#### 3.1.5. The Effect of Ultrasound Power

The effect of ultrasound power was investigated in the range of 200–800 W. Other extraction parameters were kept constant as follows: ethanol at 60%, LS ratio at 25 mL/g, extraction time at 15 min, and temperature at 50 °C. As shown in Figure 1e, the yield of antioxidants from RSB seed coats slowly increased with the increase of ultrasound power from 200 to 400 W, and then the antioxidant activity remained constant up to 800 W. This result could be due to the fact that the high ultrasound power enhanced cavity collapse, which facilitated the disintegration of cells and the release of target compounds. On the other hand, in some cases, the further increase of ultrasound power might have induced the degradation of antioxidants in the extract, adversely affecting the intensity of cavitation [34,35]. Therefore, 400 W was selected as the optimal ultrasound power.

### 3.2. Response Surface Methodology

#### 3.2.1. Response Surface Design and Experimental Results

Based on the results of the single-factor experiments, the ethanol concentration, extraction time, and LS ratio were important parameters that affected the extraction yield of antioxidants from RSB seed coats. Thus, these parameters were selected as independent variables for RSM optimization, while other extraction conditions were kept at the optimal level (temperature at 50 °C and ultrasound power at 400 W). A three-factor five-level central composite design was used in RSM optimization. The ethanol concentration at 60%, LS ratio at 25 mL/g, and extraction time at 15 min were chosen as the zero level. The detailed extraction design and the antioxidant activity of extracts (presented as TEAC values) are summarized in Table 1, with the antioxidant activity ranging from 562.01 to 750.10 μmol Trolox/g.

#### 3.2.2. Fitting the Model

Multiple regression analysis was applied to the experimental results. The response and three independent variables (coded values) were analyzed by a second-order polynomial model.

Y = 734.74 + 6.88X_1_ + 25.61X_2_ + 21.93X_3_ − 13.19X_1_X_2_ + 9.41X_1_X_3_ + 7.00X_2_X_3_ − 52.36X_1_^2^ − 17.47X_2_^2^ − 20.47X_3_^2^, (2)

where Y is the TEAC value of the extracts, and X_1_, X_2_, and X_3_ are ethanol concentration, LS ratio, and extraction time, respectively.

The results from the ANOVA of the response surface quadratic model are presented in Table 2. A high model F-value (F = 32.18), as well as a low *p*-value (*p* < 0.0001), implied the statistical significance of the model. In addition, both the determination coefficient (*R*^2^ = 0.97) and adjusted determination coefficient (Adj *R*^2^ = 0.94) were higher than 0.8, implying a high degree of correlation between the actual and predicted TEAC values. There was no significance (*p* > 0.05) for lack of fit, confirming the validity of the model. Meanwhile, the data showed that linear (X_2_ and X_3_), interactive (X_1_X_2_), and quadratic (X_1_^2^, X_2_^2^, and X_3_^2^) coefficients were significant model terms, as indicated by the *p*-values (*p* < 0.05). Other coefficients (X_1_, X_1_X_2_, and X_1_X_3_) were found to be not significant (*p* > 0.05). 

#### 3.2.3. Model Analysis

**Figure 2:**
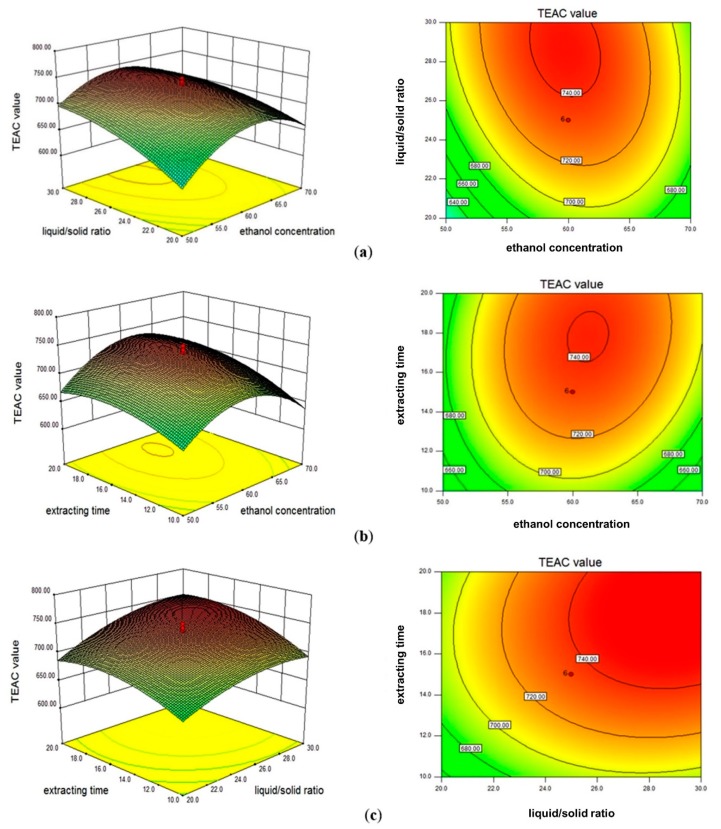
Response surface analysis showing the interactive effects of parameters, including (**a**) LS ratio and ethanol concentration, (**b**) extraction time and ethanol concentration, and (**c**) extraction time and LS ratio, on the TEAC values.

Figure 2 shows the three-dimensional surface and contour plots of the model, which allowed visualizing the effects of the three selected parameters on the TEAC values of the extracts. As shown in Figure 2a, the interactive effects between LS ratio and ethanol concentration were significant, when the extraction time was fixed at 15 min (zero level). TEAC value markedly increased as LS ratio increased from 20 to 30 mL/g, especially when the LS ratio and ethanol concentration were at low levels. When ethanol concentration was higher than about 60%, TEAC value slowly increased and then slightly decreased as LS ratio increased. In Figure 2b, the LS ratio was kept at 25 mL/g as the zero level. Both extraction time and ethanol concentration exhibited quadratic effects on the TEAC value, suggesting that there was an optimal value for each parameter in the selected range. However, the effect of extraction time was much weaker than that of ethanol concentration. In addition, Figure 2c shows that the interactive effects of extraction time and LS ratio were not significant. The response presented a similar trend as shown in Figure 2a,b. When ethanol concentration was kept at 60%, the TEAC value increased slowly as the extraction time and LS ratio increased, and the effect of the LS ratio was stronger than that of extraction time. Therefore, according to the results of ANOVA and three-dimensional surface plots, ethanol concentration was the most important parameter that affected the response, followed by LS ratio, and extraction time.

#### 3.2.4. Verification of the Optimal Extraction Condition

According to the equation and one-way ANOVA analysis, the optimal extraction conditions were as follows: ethanol at 60.2%, LS ratio at 29.3 mL/g, extraction time at 18.4 min, extraction temperature at 50 °C, and ultrasound power at 400 W. Based on these optimized parameters, the TEAC value was predicted as 753.41 μmol Trolox/g. To evaluate the validity of the RSM model, the actual TEAC value of the extract was determined under the optimal conditions. The actual TEAC value was 755.98 ± 10.23 μmol Trolox/g, which was in the predicted range. Therefore, the response model was reliable and could be used for the prediction of antioxidant yield within the range of selected extraction parameters. 

#### 3.2.5. Total Phenolic and Flavonoid Contents of RSB Seed Coats

In addition, the total phenolic and flavonoid contents of RSB seed coats under the optimal extraction conditions were 59.62 ± 2.77 mg GAE/g DW and 4.46 ± 0.15 mg CE/g DW, respectively. As an important type of natural antioxidant, polyphenols are widely present in plants, such as edible beans. It was reported that the total phenolic and flavonoid contents in common edible beans could reach 9.6 mg GAE/g DW and 4.54 mg CE/g DW, respectively [8]. The RSB seed coats contained relatively high contents of polyphenols and flavonoids compared to other beans, which might contribute to its high antioxidant capacity.

#### 3.2.6. Analysis of Main Antioxidant Polyphenols in RSB Seed Coats

The determination of main antioxidant polyphenols in RSB seed coats was carried out using HPLC–DAD–MS/MS. Five main phenolic compounds were identified and quantified from RSB seed coats (Table 3). RSB seed coats contained a high content of gallic acid, as well as its derivatives. The content of digalloyl hexoside was the highest, followed by methyl gallate, gallic acid, trigalloyl hexoside, and digallic acid. Previous reports showed that these compounds had high antioxidant activity [36], which might contribute to the strong antioxidant capacity of RSB seed coat extracts. Also, several studies found that gallic acid had many other bioactivities, such as hepatoprotective, anti-microbial, anti-inflammatory, and anticancer activities, which might be attributed to its antioxidant activity [37,38,39]. Therefore, the extracted antioxidant polyphenols in RSB seed coats could assist the further investigation of other health benefits of RSB seed coats.

#### 3.2.7. Comparison of Extraction Methods

The extraction efficiency of the UAE method was compared with that of the maceration and Soxhlet methods (Table 4). Compared with maceration, UAE treatment improved extraction efficiency by 35% with less extraction time. In comparison with Soxhlet extraction, the UAE method improved the extraction efficiency by 58% under a relatively short extraction time and low temperature, suggesting that UAE is superior to conventional maceration and Soxhlet methods to extract antioxidants from the seed coats of RSB, consistent with the results of other studies [32,40,41]. It should be pointed out that it is very difficult to strictly compare the extraction efficiency of different methods. If the same conditions are used in three extraction methods, these conditions are not all optimal conditions for each method. On the other hand, if the optimal conditions of each method are used in the three extraction methods, these conditions would not be the same. Thus, these two aspects were considered, and the literature was also referred to in this study [33,42,43].

## 4. Conclusions

In this study, the extraction conditions for antioxidants from RSB seed coats using UAE were investigated and optimized. The results from RSM showed that the antioxidant activity of RSB seed coat extracts was mainly affected by ethanol concentration, extraction time, and liquid/solid ratio. The optimal UAE conditions were as follows: 60.2% ethanol, LS ratio of 29.3 mL/g, extraction temperature of 50 °C, extraction time of 18.4 min, and ultrasound power of 400 W. Under these conditions, the antioxidant activity, total phenolic content, and total flavonoid content of the extract were 755.98 ± 10.23 μmol Trolox /g, 59.62 ± 2.77 mg GAE/g DW, and 4.46 ± 0.15 mg CE/g DW, respectively. The antioxidant activity and polyphenol contents were higher than those obtained using the maceration and Soxhlet extraction methods. In addition, the main antioxidant polyphenols in the RSB seed coat extracts were digalloyl hexoside (15.30 ± 0.98 mg/g DW), methyl gallate (8.85 ± 0.51 mg/g DW), gallic acid (8.76 ± 0.36 mg/g DW), trigalloyl hexoside (4.27 ± 0.21 mg/g DW), and digallic acid (2.89 ± 0.13 mg/g DW). Therefore, the antioxidant polyphenols obtained using UAE could be developed into functional foods or nutraceuticals with the potential to prevent and manage certain oxidative stress-related chronic diseases.

## Figures and Tables

**Table 1:** Response surface design and Trolox equivalent antioxidant capacity (TEAC) values of the extracts. DW—dry weight.

Run	X_1_ (%)	X_2_ (mL/g)	X_3_ (min)	Y (μmol Trolox/g DW)
1	1 (70)	1 (30)	−1 (10)	636.57
2	1 (70)	−1 (20)	−1 (10)	622.94
3	0 (60)	0 (25)	0 (15)	743.16
4	1 (70)	−1 (20)	1 (20)	667.62
5	−1 (50)	−1 (20)	−1 (10)	600.60
6	0 (60)	0 (25)	0 (15)	750.10
7	0 (60)	1.68 (33.4)	0 (15)	710.77
8	0 (60)	−1.68 (16.6)	0 (15)	634.98
9	0 (60)	0 (25)	0 (15)	737.80
10	0 (60)	0 (25)	−1.68 (6.6)	624.60
11	−1.68 (43.2)	0 (25)	0 (15)	562.01
12	0 (60)	0 (25)	0 (15)	723.58
13	0 (60)	0 (25)	1.68 (23.4)	704.19
14	0 (60)	0 (25)	0 (15)	729.93
15	0 (60)	0 (25)	0 (15)	728.16
16	1 (70)	1 (30)	1 (20)	712.37
17	−1 (50)	−1 (20)	1 (20)	610.75
18	−1 (50)	1 (30)	1 (20)	705.13
19	−1 (50)	1 (30)	−1 (10)	670.09
20	1.68 (76.8)	0 (25)	0 (15)	586.38

**Table 2:** The ANOVA analysis for the response surface quadratic model. df—degrees of freedom.

Effects	Source	Sum of Squares	df	Mean Square	F-Value	*p*-Value Prob > F
Total effect	Model	63,299.95	9	7033.33	32.18	<0.0001 ^a^
Linear effect	X_1_	646.04	1	646.04	2.96	0.1163
	X_2_	8954.26	1	8954.26	40.97	<0.0001 ^a^
	X_3_	6569.21	1	6569.21	30.06	0.0003 ^a^
Interactive effect	X_1_X_2_	1390.95	1	1390.95	6.36	0.0302 ^a^
	X_1_X_3_	708.40	1	708.40	3.24	0.102
	X_2_X_3_	392.06	1	392.06	1.79	0.2101
Quadratic effect	X_1_^2^	39,505.70	1	39,505.70	180.75	<0.0001 ^a^
	X_2_^2^	4397.55	1	4397.55	20.12	0.0012 ^a^
	X_3_^2^	6036.71	1	6036.71	27.62	0.0004 ^a^
	Residual	2185.65	10	218.57		
	Lack of Fit	1681.82	5	336.36	3.34	0.106
	Pure Error	503.83	5	100.77		
	Corrected Total	65,485.61	19			
	*R* ^2^	0.97				
	Adjusted *R*^2^	0.94				

^a^ Stands for statistical significance (*p* < 0.05).

**Table 3:** The content of main antioxidant polyphenols in red sword bean (RSB) seed coats.

Antioxidant Polyphenols	Retention Time (min)	Molecular Ion M^−^ (*m/z*)	MS^2^ Fragment Ions (*m/z*)	Content (mg/g DW)
Digalloyl hexoside	24.9	483	331, 313, 271, 241, 211, 179, 169, 125	15.30 ± 0.98
Methyl gallate	17.0	183	124	8.85 ± 0.51
Gallic acid	8.0	169	125	8.76 ± 0.36
Trigalloyl hexoside	40.3	635	483, 465, 331, 313, 271, 169, 125	4.27 ± 0.21
Digallic acid	29.6	321	169, 125	2.89 ± 0.13

**Table 4:** Comparison between ultrasound-assisted extraction (UAE) and conventional extracting methods.

Extraction Methods	Ethanol Concentration	Time	Temperature	TEAC Value (μmol Trolox/g DW)	TPC (mg GAE/g DW)	TFC (mg CE/g DW)
UAE	60.2%	18.4 min	50 °C	755.98 ± 10.23 ^a^	59.62 ± 2.77 ^a^	4.46 ± 0.15 ^a^
Maceration	60.2%	24 h	30 °C	558.77 ± 14.42 ^b^	40.78 ± 3.17 ^b^	2.74 ± 0.14 ^b^
Soxhlet	60.2%	4 h	95 °C	479.81 ± 12.75 ^c^	31.52 ± 1.20 ^c^	1.97 ± 0.08 ^c^

Different superscript lowercase letters in the same column indicate statistical significance at *p* < 0.05. Abbreviations: TEAC, Trolox equivalent antioxidant capacity; TPC, total phenolic content; TFC, total flavonoid content; GAE, gallic acid equivalent; CE, catechin equivalent; DW, dry weight.
